# Current News and Opinion

**Published:** 1887-01

**Authors:** 


### Current News and Opinion.

### EIGHTEENTH ANNIVERSARY OF THE FIRST DISTRICT DENTAL SOCIETY OF THE STATE OF NEW YORK.

### OFFICIAL PROGRAMME.

*Monday Evening, January* 17, 1887.

*Prayer* .............................Brady E. Backus, D. D., New York.
*Address of Welcome*.............................Dr. Wm. Carr, New York.
*Response*.............................Dr. J. Taft, Cincinnati, Ohio.

### PAPER.

James Truman, D. D. S., Philadelphia. Professor of Dental Pathology, Therapeutics and Materia Medica, University of Pennsylvania. Subject: "The Rational Basis of Practice."

*Tuesday Evening, January 18th.*

### PAPERS.

Norman W. Kingsley, D. D. S., New York. Subject: "Critical Essay on Treatment of the Irregularities of the Teeth."

J. Rollo Knapp, D D. S, New Orleans, La. Subject: "Crown and Bridge-Work."

During the Anniversary, short and interesting Papers will be read by the following gentlemen:

Frank French, M D. S., Rochester, N. Y. Subject: "What caused it?"

A. P. Southwick, M. D. S., Buffalo, N. Y. Subject: "Imperfections in Vulcanite Work."

T. D. Shumway, D. D. S., Boston, Mass. Subject: "A Monograph—Science and Dentistry."

A. M. Dudley, D. D. S., Salem, Mass. Subject: "Dentistry as Practiced by the Native Dentists of China and Japan" (Illustrated.)

On Monday and Tuesday evenings, Dr. J. Rollo Knapp will exhibit his Electric Light for Illuminating the Oral Cavity.

### CLINICS.

Clinics will be held at the New York College of Dentistry, Second Avenue and 23d Street. Clinics commence at 10 A. M. and 2 P. M. The names of the Clinical Operators will be announced the previous evening, as the committee desire *them to select their day and hour.*

*Tuesday, January 18th, 10 A. M.*

Sophy E. Feltwell, D. D. S., Pittsburgh, Pa. Artificial Crown.

J. Rollo Knapp, D. D. S., New Orleans, La. Crown and Bridge-Work. Carbon and Nitrous-Oxide Blow Pipe.

E Parmly Brown, D. D. S., Flushing, Long Island. Porcelain Crown and Bridge-Work. Electric Mallet, etc., etc.

W. W. Evans, D. D. S., Washington, D. C. Artificial Work. Dylonite lined with Aluminum. Operating with Engine-Plugger. Contour and difficult Buccal Cavities.

D. A. Williams, D. D. S., Supt. Mechanical Dept. N. Y. College of Dentistry. Modified Interdental Splint.

Truman W. Brophy, M. D , D. D. S , Professor of Oral Surgery, Chicago College of Dental Surgery, will practically demonstrate the use of his Continuous Band Matrices.

W. G. A. Bonwill, D. D. S,, Philadelphia, Pa. Clinical demonstration of the use of Amalgams.

A. W. Harlan, M. D , D. D. S., Prof of Materia Medica and Therapeutics, Chicago College of Dental Surgery. Treatment of Pyorrhœa Alveolaris.

H. C. Register, M. D., Philadelphia, Pa. Uses of Compressed Air in Dental Practice, and Atomization—A means for Cleansing the Mouth and Teeth.

E. T. Starr, D. D. S., Philadelphia, Pa Detachable Bridge-Work, etc., etc. Covering Plates with Gold Foil.

S. H. Guilford, A M., D. D. S., Philadelphia, Pa , Professor of Operative and Prosthetic Dentistry, Philadelphia Dental College and Hospital of Oral Surgery, will explain and demonstrate the Use of his Matrices.

Wm. Crenshaw, D. D. S., Atlanta, Ga Contouring Bicuspids and Molars, using Electric Mallets and Perry's Separators.

Edwin T. Darby, M D . D D. S , Philadelphia, Pa , Prof. of Operative Dentistry and Dental Histology, University of Pennsylvania, will clinic Subject not given

C. F. W. Bodecker, M. D. S , D D. S , New York City, will demonstrate the Preserving, Cutting and Mounting of Microscopical Specimens.

R Walter Starr, D D. S , Philadelphia, Pa , will show the removal of a live pulp and nerve with Dental Engine drill, using an obtunder.

John H. Meyer, D D. S., New York, will put through a Continuous Gum Case, from the Impression

M. L. Rhein, M. D., D D. S , New York. Herbst Method in Conjunction with Electric Mallet. Herbst Method of making Loop-Matrices of German Silver.

C. A. Timme, D D. S , Hoboken, N. J. Herbst Method, using Wolrab Foil.

W. Irving Thayer, M. D., D D. S., Brooklyn, N. Y., will demonstrate the Use of his Trip-Hammer Plugger.

Dr. F. P. Geran, Brooklyn, N. Y. Herbst Method, etc., etc.

W. H. Atkinson, M D., D. D. S , New York. Implantation.

Olga Neymann, D. D. S., New York. Gold Filling,

Frank Abbott, M. D., New York., Prof. of Operative Dentistry in the N. Y. College of Dentistry, will demonstrate his New Automatic Mallet, with Back Action Attachment.

Dr. Stafford G Perry, New York, will demonstrate the Perry Separator.

H. A. Parr, D. D. S., New York, Specialties in Bridge and Crown Work.

Dr. George F. Reese, Brooklyn, N. Y., will practically demonstrate the use of Reese's Metal in the different branches of Dentistry.

J. N. Farrar, M. D., D. D. S., New York, will explain Regulating Devices constructed to act upon the principle of Intermittent Force.

Frank D. Gardiner, D. D. S , Philadelphia, Pa. Electric Mallet—Practical demonstration.

The S. S. White Dental Manufacturing Company has kindly consented to exhibit all their new devices and products

Dentists are invited to bring Specimens, Models, New Appliances, Instruments, etc.

Special arrangements have been made with Messrs. Mathews & Piersons, proprietors of the Sturtevant House, Broadway and 29th Street, where full board can be obtained for $3 per day; lodgings $1 per night.

For further information apply to

Dr. W. W. Walker, 67 W. 9th St., New York,
Chairman Executive Committee.

---

"FIRST DISTRICT DENTAL SOCIETY OF THE STATE OF NEW YORK."

In the December number of the Independent Practitioner, on page 712, appears a condensed report of a special meeting of this society, called to hear a paper from Dr. N. W. Kingsley, entitled "Dentistry Not a Specialty of Medicine."

After the paper was read and discussed by members present, resolutions were passed endorsing the paper, and a committee appointed to consider the establishment of an International Dental Congress in this country "at as early a date as arrangements can be made," etc.

I was not able to be present on the occasion, but find my name on the committee appointed at the meeting, and while I appreciate the honor, I certainly could not favor the convening of a Dental Congress at "an early date," as arrangements have already been made for the working of a dental section in the forthcoming Medical Congress, and the new movement suggested would tend to detract from the interest which dentists should feel for the successful presentation of their claims on the occasion. Aside from this I see no objection to the establishment of an International Dental Congress, which like our National Association might result in great benefit to our specialty.

I must, however, take exception to the idea that "dentistry is not a specialty of medicine," and can hardly imagine how dentists of extended experience can consider the duties and requirements of their specialty at the present day as outside the pale of medical recognition. Whether such is the case or not, facts remain the same, and "facts are stubborn things." Those who are familiar with the details of dental practice, and can realize the vast amount of physical suffering relieved by its ministrations, can, if it affords them any degree of morbid satisfaction, declare that dentistry has "no recognition!"

Well, suppose they do? When dentists, *as a body*, become thoroughly fitted for the great work before them, they need have no fear that their labors will not be appreciated, nor that their specialty will fail to obtain such "recognition" as their beneficiary services to the human family entitle them to receive.

C. E. Francis.

### FIRST DISTRICT DENTAL SOCIETY.

I noticed in the Dec. Number of the INDEPENDENT PRACTITIONER, page 712, under the head of "Current News and Opinion," an article purporting to be a report of a special meeting of the First District Dental Society of the State of New York, held Nov. 19th. This meeting was called to hear Dr. Kingsley read a paper entitled "Dentistry Not a Specialty in Medicine." The report states that this was the largest and most enthusiastic meeting ever held by this society, and after the sentiments advanced had been warmly endorsed by several members, a series of resolutions, offered by Dr. Dwinelle, were adopted without a dissenting voice.

In reading the report referred to, the dental profession would naturally infer that this society fully endorsed these resolutions, which is not a fact. The true state of affairs is as follows: After the resolutions were offered and passed, the President appointed the committee of ten, referred to, when Dr. W. W. Walker stated that, as this was a special meeting, the resolutions were out of order, for they could be adopted only at a regular meeting. After some discussion the President decided that they were not in order. An appeal was then taken from his decision, but the society sustained him. At the following regular meeting of the society, the minutes and resolutions of the special meeting were laid upon the table. It is therefore evident that, as yet, the First District Dental Society has taken no action in the matter.

A MEMBER.

---

### DENTAL SUGGESTIONS.

RECUTTING ENGINE BURS.—Use a No. 2 safe-sided, fine, single-cut separating file, ground (on a corundum stone) to a feather edge from the safe or uncut side, at an angle of about 40 degrees. The temper of the bur may be drawn by holding the bur end in a coal fire till a dull red heat is produced, then dropping it on the hot part of the stove to cool slowly. If a pair of 5 or 6 inch focal distance (No. 5 or 6) spectacles be used, and the bur be placed in their focus, it will appear like the bur of a coffee mill, and the cutting with the prepared file can be easily done. For tempering, a small alcohol lamp may be used. The outer surface of the flame only produces heat. To prevent burning the steel point, the shank needs some heating before the bur is raised to the proper temperature. By a little dexterity the bulb part only needs to be heated to a cherry red, and instantly dropped into a small vessel filled with water propped up quite close to the flame. The immersion must, in small instruments, be instantaneous, or the temper required will not be obtained. No drawing of temper is required. Sandpapering, to brighten the shanks, makes them ready for use.

Skirt-braid loosely twisted before joining the ends, makes an excellent engine cord.

Emery cloth may be cut into strips for polishing fillings with a pair of dime cast iron shears, which may be sharpened on corundum stones. Their hardness makes them really better than the high priced article.

Thin slices of cork make excellent coverings for exposed pulps. They are non-conductors of heat and cold, durable and pliable.

I have heard intelligent physicians and surgeons condemn the use of the tooth brush, on the ground that it wears away the enamel of the teeth. When enamel is soft and eroded it needs removal to save the parts underneath. Good enamel will not be worn away by the brush.

In pivoting teeth, amalgam has been recommended to close the joint between the root and the porcelain To be useful, amalgam should be thoroughly incorporated into a uniform mass just where it is needed, which in setting pivot teeth cannot usually be done When it is not well worked, it will be a broken, porous mass, a great deal more deleterious than the vilest of dirt.

JOHN D. WINGATE, D. D. S.

---

### LOUISIANA STATE DENTAL SOCIETY.

The annual meeting of the Louisiana State Dental Association will be held in Tulane Hall, at New Orleans, La , on the 23d, 24th and 25th of Feb. 1887.

A cordial invitation is extended to the members of the profession throughout the States to attend. No efforts will be spared to make our guests welcome and comfortable, and the meeting interesting and profitable. An opportunity to witness the Mardi-Gras festivities will be afforded those who come, and favorable R. R. rates may be had at that time. Mardi-Gras takes place the day before the meeting. For further information address

P. J. FRIEDRICHS, Ch'm. Ex. Com.,
155 Carondelet St., New Orleans, La.

---

### MASSACHUSETTS DENTAL SOCIETY.

The twenty-second annual meeting of this Society was held in the Y. M C. A. Building, Boston, Dec. 9 and 10, 1886. The annual address was by Dr. Thos. Fillebrown. Subject: "The Influence of Culture upon Professional Skill." The officers elected were:

*President*—Dr. E. B. Hitchcock, Newton.

*First Vice-President*—Dr. H. C. Merriam, Boston.

*Second Vice-President*—Dr. G. A. Gerry, Lowell.

*Secretary*—Dr. Geo. F. Eames, Boston.

*Treasurer*—Dr. E. Page, Charlestown.

*Librarian*—Dr. R. R. Andrews, Cambridge.

*Executive Committee*—Dr. S G. Stevens, Boston; Dr. D. M. Clapp, Boston; Dr. E E. Hopkins, Boston; Dr. W. E Page, Boston; Dr. A H. Gilson, Boston.

G. F. EAMES, Secretary, 62 Trinity Terrace, Boston.

### CHICAGO DENTAL SOCIETY.

At a meeting of the Chicago Dental Society, held Dec. 7, 1886, the resolutions offered and adopted by the "First District Dental Society of the State of New York," relative to the formation of an "International Dental Congress," were endorsed by the society, and the following committee has been appointed to co-operate with the committee from the First District Dental Society: Drs. T. W. Brophy, Geo. H. Cushing, A. W. Harlan, E. D. Swain, C. F. Matteson, J. G. Reid, W. B. Ames, J. A. Swasey, N. Nelson C. N. Johnson.

J. G. REID, Rec. Sec.

Relation of Medical Men to Dental Quackery.—We have had a matter of professional etiquette referred to us lately. A medical man writes to us saying that he was called in upon two occasions to administer chloroform to patients by a man who practices dentistry, but who, he has since been informed, is not registered, and our correspondent wishes to know what course he should pursue in future. There can be only one possible answer: He should absolutely refuse to attend. To associate in any way with one who is liable to prosecution, should he ever call himself a dentist and yet practice, must bring discredit not only upon himself, but upon the medical profession generally. We will go further, and say that no medical man should allow his name to be connected in any way with dental quackery or advertisement. Dental specialists have for years worked hard, and with success, to raise their professional and social status, and they look to their parent—the medical profession—to help them by not encouraging irregular dental practitioners.—*Lancet.*

---

Miss Kate Field tells the following illustration of one of the benefits of cremation. A lady, visiting some friends, neglected to bring her tooth powder. Looking about her bed chamber she noticed an elegant vase. On removing the cover, she found a gray calcareous powder. This she regarded as dentifrice, and proceeded to avail herself of the discovery, finding it very satisfactory. The next day she mentioned the fact to her hostess, apologizing for making free with her tooth powder The countenances of the family expressed various emotions, which at last found vent in the gasp of one of the daughters: "Why, that's Aunty." Thus, as a tooth-powder, the ashes of the cremated are a success.

—*The American Lancet.*

---

The Minneapolis Dental Society has issued a very attractive programme for the year. Its officers are:

*President*—Dr. J. H. Martindale.
*Vice-President*—Dr. F. H. Brimmer.
*Secretary*—Dr. J W. Penberthy.
*Treasurer*—Dr. Loughridge.
*Librarian*—Dr. C. M. Bailey.
*Executive Committee*—Drs. E. H. Angle, H. A. Knight, and E. B. Dillingham.
*Membership Committee*—Drs. M. G Jenison, C. M Bailey, and Wm. Jermain.

---

The Illinois State Dental Society, at its last meeting, appointed a committee to aid in the formation of district societies for the State. That committee now reports, and recommends that four local associations, to be called respectively, the Northern, Eastern, Western, and Southern societies, be formed, and they publish the names of a number of dentists in each district who have promised to sustain the local association. We may, therefore, look upon the thorough organization of the dentists of Illinois as a thing already accomplished.

---

In the last five years Billroth has performed operations on the stomach in thirty two cases, with nineteen fatal results. Out of fifteen gastrectomies for cancer, there were seven recoveries.—*Med. and Surg. Reporter.*

When it is desired to solder a piece that has been soldered in another place, most gold workers consider it necessary to use a softer solder, which shall flow at a lower temperature than that first used, that the unsoldering of the previous work may be avoided. This is needless, if the solder used in the second case be placed in mercury until the surface is slightly amalgamated. If it be then used it will flow very readily, while the appearance of the finished piece is not injured, as the mercury is sublimated in the heating, leaving the solder as it originally was.

---

Tin and gold combined make an excellent filling. It may not be generally known that they unite, and in the course of time make a very hard alloy. If they be heated above the melting point of the tin, they will combine at once. Metallurgists know that tin and gold cannot be left in contact without great changes ensuing. If a bit of pure tin be placed upon a piece of gold plate and heated with the blow-pipe, it will go through the latter, leaving a hole. If the tin be melted in contact with gold, it will make the latter so brittle as to break upon slight pressure.

---

The resolutions concerning the formation of an International Dental Congress, published in this journal last month, were primarily offered and adopted at a union convention of the Fifth, Sixth, Seventh and Eighth District Dental Societies, held in Rochester, in October last. The committee appointed at that time consisted of Dr. S. B. Palmer, of the Fifth; Dr. A. M. Holmes, of the Sixth; Dr. Frank French of the Seventh; and Dr. A. P. Southwick, of the Eighth District Society.

C. T. Howard,
Rec. Sec.

---

The London Correspondent of the *N. Y. Times*, writes: "The American dentist has become almost as fixed an institution in England as the French hairdresser or the German waiter. There are probably two-score in London alone, commanding a patronage which would open the eyes of their professional brethren at home I think dentistry is probably the only thing in which Englishmen would unanimously concede American supremacy."

---

The late editor of the *London Lancet* requested that the *Lancet* should contain the following confession of his faith in connection with the notice of his death: "Feeling my deep responsibility to God for the position in which, in His Providence, He has placed me, I desire to testify to the comfort derived during my sickness from a lively faith in our Lord Jesus Christ, and that I die in the sure hope of a glorious resurrectiou."

---

The Journal of Cutaneous and Venereal Diseases will, with its January number, change its name to *The Journal of Cutaneous and Genito-Urinary Diseases*, and its scope will be broadened that much. At the same time it will be enlarged. Under the exceedingly able editorship of Prince A. Morrow, A. M., M. D, it has taken a high rank, and if it is to be improved yet more, there is no knowing where its onward march will stop.

THE PROFESSIONAL NOVEL, "Thurley Tighe," which has for two years been appearing as a kind of addendum to the *Dental Record*, of London, is at last brought to a close, all the good characters being married and happy. The story has for its author Felix Weiss, L D. S., whose name is well known in the dental literature of England, as the author of "Vernon Galbray," "The Crossing Sweeper," "Truth and Perseverance," etc., etc. "Thurley Tighe" will soon be published as a separate volume, by the Dental Manufacturing Co. of London.

---

IT IS SAID that Rome has a more bountiful supply of water than any other city in Europe, there being 591 litres in the twenty-four hours for each inhabitant. Next comes London with 300 litres for each inhabitant. Paris comes third, with 227 litres; then Naples, 200; then Berlin, with 140; sixth, Vienna with 10[illegible]; and seventh, Turin with 98.—*Med. Rec.*

---

M. JAVAL, at the late Paris Ophthalmic Congress, reported a series of cases of dental disturbance cured by operating for glaucoma. That disturbances of the dental nerves would precipitate attacks of glaucoma has been well known, but the reverse has been less considered.

---

THE DENTAL PRACTITIONER, of Philadelphia, announces that the December number is the last which will be issued Henceforth there will be no excuse for the continuance of the practice of certain dental journals, to borrow largely from THE INDEPENDENT PRACTITIONER and then give credit to *The Dental Practitioner.*

---

SOZODONT. According to the *American Analyst*, this tooth-wash consists of soap, 5 parts; glycerine, 6 parts; spirits, 30 parts; water, 20 parts Flavored with several cheap oils, and colored. The accompanying tooth-powder is a mixture of orris root, chalk and magnesia

---

RESEARCHES BY DR NEWTON, published in the *Medical News*, prove that milk warm from the cow, when placed in tight cans in a warm atmosphere, will so change as to develop poisonous ptomaines, sufficient to cause toxic symptoms in those using the milk.

---

DR. WEIR MITCHELL, of Philadelphia, is said to have written a second novel. Dr. W. A. Hammond, of New York, is the author of several novels. Medical novelists are on the increase —*Maryland Medical Journal.*

---

"WE ARE never afraid of the man who keeps his mouth open," says the shrewd Indian. A closed mouth indicates power not to be lightly esteemed

---

"MRS. MULCAHEY, have you heard of the great rimidy for hydrophoby ?" "No, faith And phat is it ?" "Plasteur of Paris, sure "

---

THERE ARE 5,237 insane patients in New York City institutions.

---

THERE ARE but three free dispensaries in Paris.

